# Association between *CYP2B6* polymorphism and acute leukemia in a Han population of Northwest China

**DOI:** 10.1002/mgg3.1162

**Published:** 2020-03-02

**Authors:** Li‐Li Yu, Wei Zhang, Juan Li, Li Zhao

**Affiliations:** ^1^ Department of Oncology Lanzhou University Second Hospital Lanzhou China; ^2^ Centre Laboratory Lanzhou University First Hospital Lanzhou China

**Keywords:** acute leukemia, allele, *CYP2B6*, genotype, Han Chinese, polymorphism

## Abstract

**Background:**

The aim of this study was to investigate potential associations between *CYP2B6* c.516G>T polymorphism and the occurrence and prognosis of acute leukemias (AL) in the Han population of Northwest China.

**Methods:**

The *CYP2B6* gene polymorphism was analyzed by PCR‐RFLP and Sanger DNA sequencing in 126 patients with AL and 161 healthy controls.

**Results:**

Compared with controls, there were significantly higher frequencies of GT and TT genotypes and T alleles in AL patients (*p* < .05), particularly in fusion gene‐positive AL patients. There was no significant difference in *CYP2B6* polymorphic genotypes and T alleles between AL patients with complete remission after the first course of chemotherapy and controls (*p* > .05), while the frequencies in AL patients with partial remission and no remission were significantly higher. The *CYP2B6* allele frequency in Han Chinese in Northwest China was significantly different to that reported in Han Chinese and other ethnic minorities in southern China, Uygur Chinese, Vietnamese, African, German, British, Spanish, Turkish, and Argentinian populations; however, there was no significant difference compared with allele frequencies reported in Tibetan and Mongolian Chinese, Japanese, Korean, and American populations.

**Conclusion:**

Our findings show a strong correlation of the *CYP2B6* c.516G>T polymorphism in the Han population of Northwest China with AL, especially fusion gene‐positive AL, and indicate a poor prognosis after the first course of chemotherapy. Our findings also implicate the T allele in AL susceptibility and indicate the existence of racial and geographical differences in allele frequencies of *CYP2B6* c.516G>T polymorphism.

## Graphical abstract

We investigated associations of *CYP2B6* c.516G>T polymorphism with acute leukemias (AL) in Han Chinese. *CYP2B6* polymorphic genotypes and T alleles were associated with fusion gene‐positive AL. These AL patients also had a poor prognosis after the first course of chemotherapy.

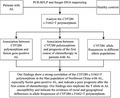

## 1 INTRODUCTION

Acute leukemia (AL) is a type of tumor that is relatively sensitive to environmental carcinogens. Although the etiology and pathogenesis of AL is unclear, it is generally agreed that its occurrence is related to both environmental and genetic risk factors (Berkoz & Yalin, 2009; Yuan et al., 2011).

The human cytochrome P450 (CYP) superfamily consists of phase I biotransformation and metabolism enzymes that play a critical role in the metabolism of many endogenous and exogenous compounds and drugs, and participates in the in vivo detoxification of many procarcinogens/carcinogens, teratogens, and toxic substances (He & Feng, 2015; Yin et al., 2018). The *CYP2B6* gene (OMIM#123930), which is located at 19q13.2, spans approximately 27.1 kb and contains 11 exons and 10 introns. To date, in addition to the wild‐type CYP2B6*1 allele, more than 100 single nucleotide polymorphisms (SNPs) and at least 30 different alleles of the *CYP2B6* gene have been described, including the SNP c.516G>T (Q172H; rs3745274). This polymorphism results in a guanine to thymine substitution at nucleotide 516 in exon 4 (rs3745274), and consequently, a glutamine to histidine substitution of the amino acid at position 172 (Gln172His). This missense polymorphism affects metabolic activity by altering substrate binding or aberrant splicing, leading to decreased amounts of the normal mRNA transcript, and consequently, to decreased levels of expression and function of *CYP2B6* protein, thereby blocking the conversion of carcinogens to inactive metabolites (Berkoz & Yalin, 2009).

A number of *CYP2B6* polymorphisms have been reported in the normal population, and ethnic differences have been noted. Studies have shown that *CYP* gene polymorphisms are associated with tumor genetic susceptibility (He & Feng, 2015; Yin et al., 2018). Although some studies have implicated the *CYP2B6* c.516G>T polymorphism in the development of AL, there are no relevant reports related to the Han Chinese population in Northwest China (Berkoz & Yalin, 2009; Daraki et al., 2014; Yuan et al., 2011).

In this study, we aimed to investigate a potential association between *CYP2B6* c.516G>T gene polymorphism in the Han Chinese population in Northwest China and the occurrence and prognosis of AL. We also compared the allele frequencies *of CYP2B6* c.516G>T polymorphism identified in the current study with those reported previously in Han Chinese and other ethnic minorities in southern China, Tibetan Chinese, Mongolian Chinese, Uygur Chinese, and other ethnic populations to clarify the distribution characteristics and geographical and ethnic differences in the normal population.

## 2 MATERIALS AND METHODS

### 2.1 Ethical compliance

Informed consent from patients as well as from controls was obtained. The study was approved by the Ethics Committee of Lanzhou University.

### 2.2 Study population

The patient group comprised of 126 patients with AL (73 males and 53 females; aged 3–82 years [median, 30 years]) who were treated in the Department of Hematology, the First Hospital of Lanzhou University (China), and the Center for Hematologic Diseases of Chinese PLA, Lanzhou Military Command General Hospital (China) between June 2013 and August 2016. All patients were diagnosed by bone marrow cell morphology, histochemistry, and immunophenotype according to the French–American–British (FAB) criteria for the diagnosis of AL (Sharma & Mohindroo, 2004). Among these patients, 45 were diagnosed with acute lymphocytic leukemia (ALL) and 81 with acute myeloid leukemia (AML). The control group comprised of 161 unrelated healthy individuals attending the First Hospital of Lanzhou University for a checkup only. The control group was age‐ and sex‐matched to the AL group. Demographic data of the studied population are shown in Table 1.

**Table 1:** Demographic characteristics of the AL patients and control group

	AL	Control	*p*‐value
Number	126	161	
Sex [*n* (%)]			
Males	73 (57.9%)	92 (57.1%)	.89
Females	53 (42.1%)	69 (42.9%)	
Age (years)			
Mean ± *SD*	30.35 ± 19.01	32.52 ± 13.27	.26

Abbreviations: AL, acute leukemia; *SD*, standard deviation.

### 2.3 DNA extraction

Bone morrow samples (2 ml) from AL patients and peripheral blood samples (2 ml) from the control group were collected into tubes containing EDTA. Genomic DNA was extracted by the conventional phenol–chloroform method (Poncz et al., 1982).

### 2.4 Genotyping

The GenBank reference for *CYP2B6* is NC_000019.10. *CYP2B6* c.516G>T polymorphism was genotyped by PCR‐RFLP as previously described (Berkoz & Yalin, 2009) and using previously reported PCR primers (Nakajima et al., 2007). PCR amplification was performed in a total reaction volume of 25 μl containing 100 ng genomic DNA,10 × PCR buffer, 2 mmol/L dNTPs, 25 mmol/L MgCl_2_, 0.1 mg/ml of each primer, and 1.25 units of Taq polymerase (Shanghai Sangon Biological Engineering Technology & Services). After an initial denaturation at 94℃ for 5 min, the amplification was performed by 35 cycles of denaturation at 94℃ for 30 s, annealing at 57℃ for 30 s, and extension at 72℃ for 45 s, with a final extension step at 72℃ for 7 min. A sample (5 μl) of the PCR product was then digested at 65℃ for 4 hr with 6 units of *BseN*I restriction enzyme (MBI, Fermentas). DNA fragments were separated by agarose gel (3%) (BBI) electrophoresis. Some samples were delivered to Beijing Liuhe Huada Gene Technology Co., Ltd. for Sanger DNA sequencing to confirm the PCR‐RFLP results. The BCR‐ABL and PML‐RARα fusion genes in AL patients were determined by a quantitative real‐time PCR (Q‐PCR) instrument (LC480, Roche).

### 2.5 Statistical analysis

Statistical analyses were performed using the SPSS 19.0 software (IBM Corporation). Genotype and allele frequencies were calculated by direct counting. Hardy–Weinberg equilibrium was carried out by comparing the observed and expected genotype frequencies for the *CYP2B6* gene polymorphism using a chi‐square test. Differences in genotype and allele frequencies between cases and controls were evaluated by the chi‐square test. The relative risk was expressed as the odds ratio (OR) and 95% confidence intervals (CI). Chi‐square tests were also used to examine differences in the distributions of *CYP2B6* allele frequencies identified in the current study and those reported previously (Arenaz et al., 2010; Cho et al., 2004; Guan et al., 2006; Haas et al., 2004; Hiratsuka et al., 2002; Jacob, Johnstone, Neville, & Walton, 2004; Kirchheiner et al., 2003; Klein et al., 2005; Lamba et al., 2003; Li et al., 2012; Qi et al., 2016; Scibona, Vazquez, Cajal, Argibay, & Belloso, 2015; Shu et al., 2017; Veiga et al., 2009; Xu et al., 2007; Yuce‐Artun, Kose, & Suzen, 2014). *p* < .05 was considered to indicate statistical significance.

## 3 RESULTS

### 3.1 PCR amplified and digestion products

**Figure 1:**
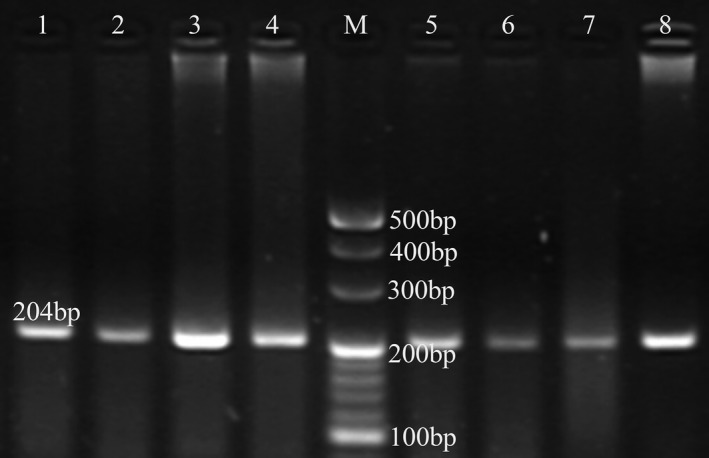
*CYP2B6* gene PCR amplification products yielded a single fragment of 204 bp (*CYP2B6*;NC_000019.10)

**Figure 2:**
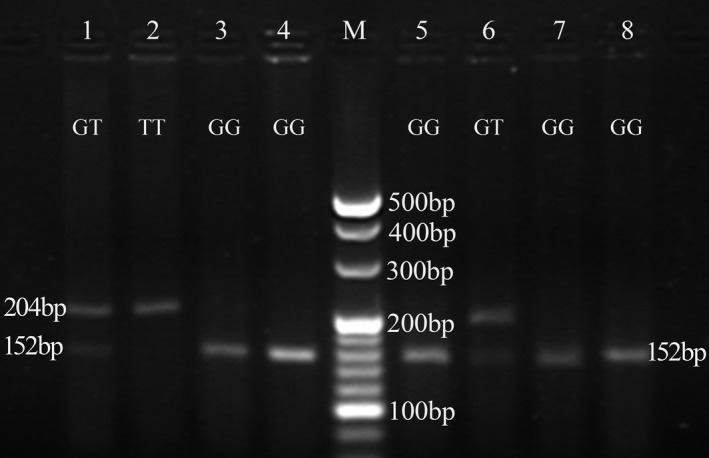
*CYP2B6* gene digestion products: GG produced a double band of 152 and 52 bp, GT produced three bands at 204, 152 and 52 bp and TT produced only one band of 204 bp (*CYP2B6*;NC_000019.10)

PCR amplification of the *CYP2B6* gene yielded a single fragment of 204 bp (Figure 1). In RFLP analysis, the wild‐type genotype (GG) produced a double band of 152 and 52 bp, heterozygotes (GT) produced three bands at 204, 152, and 52 bp, and the homozygote polymorphic genotype (TT) produced only one band of 204 bp (Figure 2).

### 3.2 The results of *CYP2B6* Sanger DNA sequencing

**Figure 3:**
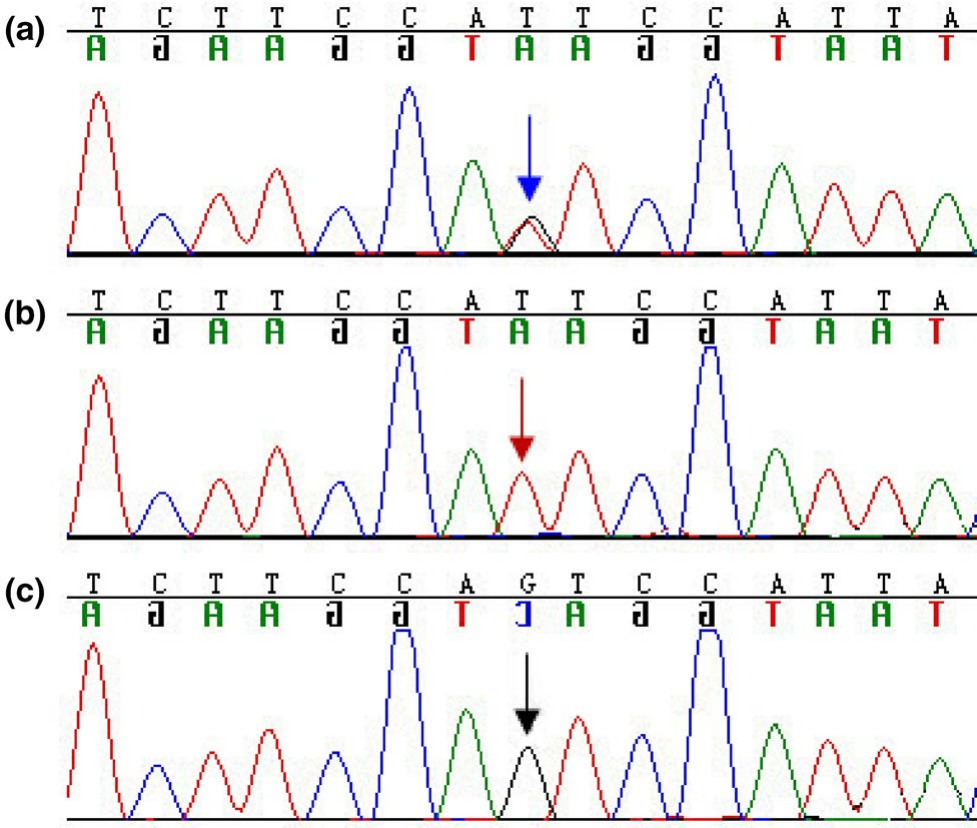
The results of *CYP2B6* Sanger DNA sequencing. (a) The blue arrow is referred to as the GT heterozygote; (b) The red arrow indicates the TT homozygote; (c) The black arrow indicates GG wild genotype (*CYP2B6*;NC_000019.10)

The reliability of the PCR‐RFLP results was further verified by *CYP2B6* c.516G>T polymorphism loci GG, GT, and TT genotype sequencing analysis (Figure 3). The blue arrow in the Figure 3a is referred to as the GT heterozygote; the red arrow in the Figure 3b chart indicates the TT homozygote; and the Figure 3c black arrow indicates GG wild genotype.

### 3.3 *CYP2B6* genetic polymorphisms

Genotype counts and allele frequencies of *CYP2B6 c*.516G>T polymorphism in the case and control groups are shown in Table 2. Three genotypes, GG, GT, and TT, at the *c*.516G>T polymorphism loci were identified in both the case and control groups.

**Table 2:** Genotype counts and allele frequencies *of CYP2B6* c.516G>T polymorphism

Groups	Cases	Genotypes [*n* (%)]			Allele frequencies (%)	
		GG	GT	TT	G	T
Control	161	124 (77.0)	25 (15.5)	12 (7.5)	84.8	15.2
ALL	45	25 (55.6)	13 (28.9)*	7 (15.5)*	70.0	30.0*
AML	81	47 (58.0)	22 (27.2)*	12 (14.8)*	71.6	28.4*

Abbreviations: ALL, acute lymphocytic leukemia; AML, acute myeloid leukemia.

a *p* < .05 versus control. The GenBank reference for *CYP2B6* is NC_000019.10.

The proportions of GT and TT genotypes at the c.516G>T polymorphism loci in ALL patients (28.9% and 15.5%, respectively) were significantly higher than those in the healthy controls (15.5% and 7.5%, respectively; *p* = .017 and .036, respectively; Table 2). The OR of ALL associated with the c.516G>T polymorphism loci GT and TT genotypes was 2.579 and 2.893, respectively.

The proportions of GT and TT genotypes at the c.516G>T polymorphism loci in AML patients (27.2% and 14.8%, respectively) were significantly higher than those in the healthy controls (*p* = .012 and .025, respectively; Table 2). The OR of AML in c.516G>T polymorphism loci GT and TT genotypes was 2.322 and 2.638, respectively.

The T allele frequencies of c.516G>T polymorphism in ALL and AML patients (30.0% and 28.4%, respectively) were significantly higher than those in the healthy controls (*p* = .001, Table 2). The OR of ALL and AML in T allele carriers was 2.388 and 2.209, respectively.

### 3.4 Association between *CYP2B6* polymorphism and fusion gene‐positive AL

The variants and T allele frequencies of *CYP2B6* c.516G>T polymorphism in BCR‐ABL fusion gene‐positive AL patients (81.8% and 54.5%, respectively) were significantly higher than those in the healthy controls (23.0% and 15.2%, respectively; *p* = .000, Table 3). The OR of variants and T alleles in BCR‐ABL fusion gene‐positive AL patients was 15.081 and 6.686, respectively.

**Table 3:** Association between *CYP2B6* c.516G>T polymorphisms and fusion gene‐positive AL

Groups	Cases	Genotypes [*n* (%)]		Allele frequencies (%)	
		GG	GT + TT	G	T
Control	161	124 (77.0)	37 (23.0)	84.8	15.2
AL					
BCR‐ABL(+)	11	2 (18.2)	9 (81.8)*	45.5	54.5*
PML‐RAR_α_(+)	11	5 (45.5)	6 (54.5)*	68.2	31.8*

Abbreviations: AL, acute leukemia; BCR‐ABL(+), BCR‐ABL fusion gene‐positive; PML‐RAR_α_(+), PML‐RAR_α_ fusion gene‐positive.

a *p* < .05 versus control. The GenBank reference for *CYP2B6* is NC_000019.10.

The variants and T allele frequencies of c.516G>T polymorphism in PML‐RARα fusion gene‐positive AL patients (54.5% and 31.8%, respectively) were significantly higher than those in healthy controls (*p* = .019 and .041, respectively; Table 3). The OR of variants and T alleles in PML‐RARα fusion gene‐positive AL patients was 4.022 and 2.600, respectively.

### 3.5 Association between *CYP2B6* polymorphism and prognosis of the first course of chemotherapy in patients with AL

There was no significant difference in the variants and T allele frequencies of *CYP2B6* c.516G>T polymorphism among the AL patients achieving complete remission (CR) after the first course of chemotherapy and the healthy controls (Table 4).

**Table 4:** Association between *CYP2B6* c.516G>T polymorphism and prognosis of the first course of chemotherapy patients with AL

Groups	Cases	Genotypes [*n* (%)]		Allele frequencies (%)	
		GG	GT + TT	G	T
Control	161	124 (77.0)	37 (23.0)	84.8	15.2
AL					
CR	63	41 (65.1)	22 (34.9)	77.8	22.2
PR	22	11 (50.0)	11 (50.0)*	61.4	38.6*
NR	41	23 (56.1)	18 (43.9)*	72.0	28.0*

Abbreviations: AL, acute leukemia; CR, complete remission; PR, partial remission; NR, no remission.

a *p* < .05 versus control. The GenBank reference for *CYP2B6* is NC_000019.10.

The variants and T allele frequencies of c.516G>T polymorphism in AL patients achieving partial remission (PR) after the first course of chemotherapy (50.0% and 38.6%, respectively) were significantly higher than those in the healthy controls (23.0% and 15.2%, respectively) (*p* = .007 and .000, respectively; Table 4). The OR of variants and T alleles in AL patients achieving PR were 3.351 and 3.508, respectively.

The variants and T allele frequencies of c.516G>T polymorphism in AL patients with no remission (NR) after the first course of chemotherapy (43.9% and 28.0%, respectively) were significantly higher than those in the healthy controls (*p* = .007, Table 4). The OR of variants and T alleles in AL patients with NR were 2.623 and 2.172, respectively.

### 3.6 *CYP2B6* allele frequencies in different ethnic populations

In this study, we compared the allele frequencies of *CYP2B6* c.516G>T polymorphism previously reported in southern China (Guan et al., 2006), ethnic minorities in southern China (Xu et al., 2007), Tibetan Chinese (Qi et al., 2016), Mongolian Chinese(Qi et al., 2016), Uygur Chinese (Qi et al., 2016), and other ethnic populations (Arenaz et al., 2010; Cho et al., 2004; Haas et al., 2004; Hiratsuka et al., 2002; Jacob et al., 2004; Kirchheiner et al., 2003; Klein et al., 2005; Lamba et al., 2003; Li et al., 2012; Scibona et al., 2015; Veiga et al., 2009; Yuce‐Artun et al., 2014) with those in 161 normal controls (Table 5). There was a statistically significant difference in the allele frequencies of *c*.516G>T polymorphism between our population of Han Chinese in Northwest China and reports of Vietnamese, Han Chinese in southern China, ethnic minorities in southern China, Uygur Chinese, African, German, British, Spanish, Turkish, and Argentinian populations (*p* < .05, Table 5); however, there were no significant differences in the allele frequencies of *c*.516G>T polymorphism between Han Chinese in Northwest China and reports of Tibetan Chinese, Mongolian Chinese, Japanese, Korean, and American populations (*p* > .05, Table 5).

**Table 5:** The allele frequency of *CYP2B6* c.516G>T polymorphism in different ethnic populations

Population	*n*	Frequency of allele *CYP2B6* c.516G>T (%)
Han Chinese in Northwest China (current study)	322	15.2
Japanese (Hiratsuka et al., 2002)	530	16.4
Korean (Cho et al., 2004)	716	14.0
Vietnamese (Veiga et al., 2009)	156	27.0*
Han Chinese in southern China (Guan et al., 2006)	386	21.0*
Ethnic minorities in southern China (Xu et al., 2007)	1,014	34.5*
Tibetan Chinese (Qi et al., 2016)	646	14.7
Mongolian Chinese (Qi et al., 2016)	268	17.9
Uygur Chinese (Qi et al., 2016)	324	28.7*
African–American (Haas et al., 2004)	100	38.0*
Ghanaians (Klein et al., 2005)	82	48.8*
West Africans (Li et al., 2012)	306	50.0*
German (Kirchheiner et al., 2003)	1,146	26.0*
British (Jacob et al., 2004)	270	28.1*
American (Lamba et al., 2003)	86	22.0
Spanish (Arenaz et al., 2010)	360	21.5*
Turkish (Yuce‐Artun et al., 2014)	344	28.0*
Argentinian (Scibona et al., 2015)	204	28.9*

Abbreviation: *n*, Total number of alleles.

a *p* < .05, Current study versus other ethnic population. The GenBank reference for *CYP2B6* is NC_000019.10.

## 4 DISCUSSION

Increasing interest in *CYP2B6* genetic polymorphism has been stimulated by revelations of a specific *CYP2B6* genotype that significantly affects the metabolism of various drugs in common clinical use in terms of increasing drug efficacy and avoiding adverse drug reactions (Yuce‐Artun et al., 2014). In recent years, the research on *CYP2B6* gene polymorphism has focused on its pharmacokinetic and pharmacodynamic effects on the chemotherapy drug cyclophosphamide (Shu et al., 2017), the anesthetics ketamine (Wang, Neiner, & Kharasch, 2018), the central nervous system‐active bupropion (Tomaz et al., 2015) and methadone (Ahmad, Valentovic, & Rankin, 2018), and the antiretroviral drug efavirenz (von Braun et al., 2018). In addition, the distribution of *CYP2B6* genotypes have been studied in several different populations (Szalai, Hadzsiev, & Melegh, 2016; Yuce‐Artun et al., 2014). Extensive interindividual variations in pharmacokinetics are considered to be a major reason for unpredictable drug responses, while individual pharmacogenetics affect the pharmacokinetics and pharmacodynamics of drugs.

Interactions of genetic polymorphism with diet, smoking, and some environmental substances can affect the outcome of AL (Brisson, Alves, & Pombo‐de‐Oliveira, 2015; Pakakasama et al., 2005). It has been reported that *XPD* Lys751Gln, *GSTM1*null, *CYP2D6*,* CYP2E1*,* NQO1*,* NAT2*,* MDR1,* and *XRCC1* polymorphisms are associated with genetic susceptibility to AL (Brisson et al., 2015; Dong et al., 2008; Lemos et al., 1999; Liu, Wu, Li, & Dong, 2014). Furthermore, studies have shown that: (a) *GSTM1*, CYP1A1*2A,* NQO1* C609T, *MTHFR* C677T, and A1298C polymorphisms are associated with the risk of ALL (Krajinovic, Labuda, Richer, Karimi, & Sinnett, 1999; Li & Zhou, 2014; Pakakasama et al., 2005; Xiao, Deng, Su, & Shang, 2014); (b) the intronic rs6021191 variant in nuclear factor of activated T cells 2 (NFATC2), *TPMT*,* MTHFR*,* SLCO1B1,* and *CYP3A4* polymorphisms are associated with toxicity of ALL‐related drugs (Fernandez et al., 2015; Lopes et al., 2004; Lopez‐Lopez et al., 2014); and (c) polymorphism in the miR‐204 flanking region and *IDH* mutations may constitute a prognostic factor in AML (Nowak et al., 2018; Xu et al., 2017).

Accumulating evidence has provided insights into the substrates, inducers, inhibitors, and gene polymorphisms of *CYP2B6*; however, potential correlations between *CYP2B6* polymorphisms and tumors, especially the occurrence and prognosis of leukemia, remain to be established. There is strong evidence indicating that *CYP2B6* polymorphisms are involved in the development not only of solid tumors, such as breast cancer (Justenhoven et al., 2014) and hepatocellular carcinoma (Yan et al., 2017), but also in hematological malignancies (Alazhary, Shafik, Shafik, & Kamel, 2015; Berkoz & Yalin, 2009; Daraki et al., 2014; Yuan et al., 2011). To date, only three studies have revealed a correlation between *CYP2B6* c.516G>T polymorphism and AL (Berkoz & Yalin, 2009; Daraki et al., 2014; Yuan et al., 2011), and one study report that the *CYP2B6 c*.516G>T polymorphism was not associated with AML prognosis (Alazhary et al., 2015). However, associations between *CYP2B6* c.516G>T polymorphism, and the occurrence and prognosis of AL in the Han population of Northwest China have not yet been reported.

In a study of the *CYP2B6* c.516G>T polymorphism in 80 patients with AL (44 patients with ALL, 36 patients with AML) and 100 healthy controls, Berkoz and Yalin (2009) found that the GT genotype was significantly associated with AL (OR = 2.481, *p* = .003); thus, indicating that GT genotypes may be an important genetic marker of AL.

In the present study, we identified three genotypes (GG, GT, and TT) of *CYP2B6* c.516G>T polymorphism in ALL, AML, and healthy controls. Our study showed that the GT and TT genotype were associated with the occurrence of ALL and AML (OR: 2.579, 2.322, 2.893, and 2.638, respectively; *p* < .05). The OR of ALL and AML in T allele carriers was 2.388 and 2.209, respectively, suggesting that the T allele may be associated with susceptibility to AL.

The BCR‐ABL fusion gene is a characteristic cytogenetic change in chronic myeloid leukemia and certain ALL patients, resulting from the fusion of the *BCR* gene of the long arm of chromosome 22 with the *ABL* gene of the long arm of chromosome 9 (Kang et al., 2016). For patients with ALL, the BCR‐ABL fusion gene often indicates a poor prognosis (Kang et al., 2016). Among the 126 patients with AL included in our study, eight cases of ALL and three cases of AML were BCR‐ABL fusion gene‐positive. Analysis of the *CYP2B6* c.516G>T polymorphism showed a strong correlation of the polymorphic genotype and T allele of c.516G>T polymorphism with BCR‐ABL fusion gene‐positive AL (OR: 15.081 and 6.686, respectively; *p* < .05).

According to statistics, more than 90% of M3 (acute promyelocytic leukemia) type AML is associated with abnormal karyotype t (15;17) (q21; q22), which forms a PML‐RARα fusion gene. This variant, which is not found in other types of primary AL, represents the molecular basis for the onset of M3 and effective treatment with all‐*trans* retinoic acid or arsenic trioxide. The 81 AML patients included in our study included 25 with M3 type and 11 of these patients were PML‐RARα fusion gene‐positive. Further analysis revealed that the polymorphic genotype and T allele of the *CYP2B6* c.516G>T polymorphism were associated with PML‐RARα fusion gene‐positive AL (OR: 4.022 and 2.600, respectively; *p* < .05).

In the present study, we found that the polymorphic genotype and T allele of *CYP2B6* c.516G>T polymorphism were not associated with CR in AL after the first course of chemotherapy. However, the polymorphic genotype and T allele of c.516G>T polymorphism were associated with PR/NR in AL after the first course of chemotherapy. These findings suggested that AL patients with the polymorphic genotype and T allele of *CYP2B6* c.516G>T polymorphism have a poor prognosis following the first course of chemotherapy.

Although the allele frequency of *CYP2B6* polymorphism has been studied in several different populations, there is no data regarding the distribution among the Han Chinese population in Northwest China. Our analysis suggested that *CYP2B6* polymorphism is associated with ethnic variation. Guan et al. (2006) showed that the allele frequencies of *CYP2B6* polymorphism in Chinese Han and Japanese, South Korean, and American populations were similar, but different from those of German and African populations. Xu et al. (2007) reported that the allele frequencies of *CYP2B6* polymorphism in ethnic minorities in southern China was significantly higher than that in all Asian populations, including Japanese, South Korean, and Chinese Han. In addition, the allele frequency was different from that found in Caucasians (German, American, Swiss, Canadian, and British). Thus, the results of our study are basically consistent with those reported by Guan et al., but are significantly different from the allele frequencies of *CYP2B6* c.516G>T polymorphism in Chinese Han and ethnic minorities in southern China, which may be related to the geographical selection of samples. Furthermore, Guan et al studied the Chinese Han population in southern China, which is mainly concentrated in Guangdong Province, while Xu et al studied three ethnic minorities in Yunnan Province: Wa, Lahu, and Blang; thus, it can be speculated that geographical differences may affect the distribution of allele frequencies.

## CONFLICT OF INTEREST

The authors declare that there is no conflict of interest.
